# Examination of Single-Walled Carbon Nanotubes Uptake and Toxicity from Dietary Exposure: Tracking Movement and Impacts in the Gastrointestinal System

**DOI:** 10.3390/nano5021066

**Published:** 2015-06-12

**Authors:** Joseph H. Bisesi, Thuy Ngo, Satvika Ponnavolu, Keira Liu, Candice M. Lavelle, A.R.M. Nabiul Afrooz, Navid B. Saleh, P. Lee Ferguson, Nancy D. Denslow, Tara Sabo-Attwood

**Affiliations:** 1 Department of Environmental and Global Health, Center for Environmental and Human Toxicology, University of Florida, Gainesville, FL 32611, USA; E-Mails: jbisesi@phhp.ufl.edu (J.H.B.); tweetiebird@ufl.edu (T.N.); sponnavolu@ufl.edu (S.P.); clavelle@ufl.edu (C.M.L.); 2 Department of Civil and Environmental Engineering, Nicholas School of the Environment, Duke University, Durham, NC 27708, USA; E-Mails: keira.liu@duke.edu (K.L.); lee.ferguson@duke.edu (P.L.F.); 3 Department of Physiological Sciences, Center for Environmental and Human Toxicology, University of Florida, Gainesville, FL 32611, USA; E-Mail: ndenslow@ufl.edu; 4 Department of Civil, Architectural, and Environmental Engineering, The University of Texas Austin, Austin, TX 78712, USA; E-Mails: afrooz@utexas.edu (A.R.M.N.A.); navid.saleh@utexas.edu (N.B.S.)

**Keywords:** single-walled carbon nanotubes(SWCNTs), fish, near-infrared fluorescence (NIRF), sorption, gastrointestinal system, gene expression, nutrient transporters

## Abstract

Previous studies indicate that exposure of fish to pristine single-walled carbon nanotubes (SWCNTs) by oral gavage, causes no overt toxicity, and no appreciable absorption has been observed. However, in the environment, SWCNTs are likely to be present in dietary sources, which may result in differential impacts on uptake and biological effects. Additionally, the potential of these materials to sorb nutrients (proteins, carbohydrates, and lipids) while present in the gastrointestinal (GI) tract may lead to nutrient depletion conditions that impact processes such as growth and reproduction. To test this phenomenon, fathead minnows were fed a commercial diet either with or without SWCNTs for 96 h. Tracking and quantification of SWCNTs using near-infrared fluorescence (NIRF) imaging during feeding studies showed the presence of food does not facilitate transport of SWCNTs across the intestinal epithelia. Targeting genes shown to be responsive to nutrient depletion (peptide transporters, peptide hormones, and lipases) indicated that *pept2*, a peptide transporter, and *cck*, a peptide hormone, showed differential mRNA expression by 96 h, a response that may be indicative of nutrient limitation. The results of the current study increase our understanding of the movement of SWCNTs through the GI tract, while the changes in nutrient processing genes highlight a novel mechanism of sublethal toxicity in aquatic organisms.

## 1. Introduction

Numerous industrial and commercial uses of single-walled carbon nanotubes (SWCNTs) have resulted in a heavy expansion of production of these materials [1,2]. Aquatic environments often act as sinks for both point and non-point source contaminants, therefore it is likely that SWCNTs are making their way to these systems through manufacturing waste streams and leaching. As a result, there is a growing need to characterize potential impacts of SWCNTs on aquatic environments.

Studies that have examined the toxicity of SWCNTs to fish have found that these materials, in general, do not cause overt toxicity. While there is some evidence that waterborne exposures to these materials may cause respiratory stress [3], oral exposures through gavage and feeding have produced minimal responses [4,5]. But one of the biggest barriers to comprehensive toxicity assessment including uptake, distribution, and sublethal toxicity of SWCNTs, has been the difficulty in detection and quantification of these materials *in vivo*. Attempts to quantify the distribution of these materials have employed such methods as transmission electron microscopy (TEM), Raman spectroscopy, fluorescent tagging, thermogravimetric analysis, size exclusion chromatography, and absorbance measurements. Each of these methods has its own set of limitations, some of which include complicated and time consuming sample preparation, low sensitivity, and heavy modification of materials [6,7,8,9,10]. The more recent application of near-infrared fluorescence (NIRF) for imaging and quantification of SWCNTs has emerged as a rapid and sensitive technique to assess the distribution of these materials in cells and whole organisms [5,11,12,13]. This technique takes advantage of the fluorescence in the near-infrared range (900–1500 nm) of semi-conducting SWCNTs with specific chiral wrapping vectors (*n*, *m*) when excited with 600–800 nm wavelength lasers [14,15]. This application is of particular interest for *in vivo* assessments as biological tissue does not typically exhibit endogenous background fluorescence in the near-infrared [16]. As a result of these distinct advantages, NIRF imaging and quantification has been successfully demonstrated as a sensitive technique to examine distribution and effects of SWCNTs in fish through oral gavage exposures [5]; however, further studies are needed to determine the effectiveness of this technique in more environmentally relevant exposure scenarios.

The hydrophobic nature of SWCNTs indicates that these materials are unlikely to remain suspended in aquatic systems but will more commonly sorb to sediments or biota [12]. As a result, these materials are expected to enter aquatic food chains through dietary routes. Yet very few studies have examined dietary exposures of aquatic organisms to SWCNTs. In rainbow trout (*Oncorhynchus mykiss*) exposure to SWCNTs via dietary routes did not exhibit any direct effects on survival, growth, ion regulation, biochemistry, and histopathology, but did show impacts on measures of oxidative stress in brain tissues [4]. While it seems that the direct impacts of SWCNTs consumed through diet are likely minimal, it is quite possible that these materials may indirectly affect aquatic organisms through interactions with nutritional and chemical constituents in the diet. It has been demonstrated in numerous studies that the hydrophobic nature of SWCNTs leads to high sorption affinity for proteins and lipids from suspension environments [17,18,19]. Therefore it was hypothesized that interactions of SWCNTs and nutritional components present in fish diet will cause alterations in genes responsible for nutrient transport and processing.

It has previously been demonstrated that nutrient depletion or starvation of fish leads to differential expression of genes involved in nutrient processing. For example, the intestinal peptide transporters *pept1* and *pept2* have been shown to be responsive to starvation and re-feeding and are thought to play a role in compensatory growth [20,21]. The peptide hormone cholecystokinin (*cck*), which plays a role in stimulating digestion and signaling satiation, has been found to be differentially expressed seasonally as well as during periods of fasting [22]. Alterations in dietary lipids have been found to impact the expression of lipoprotein lipase (*lpl*), an enzyme responsible for digestion of specific lipids [23,24]. As a result of their responsiveness to nutrient depletion, these genes are ideal candidates for examining the impacts of SWCNTs on nutrient depletion in fish.

The objectives of this study were twofold: (1) to determine if SWCNTs present in the diet influence the uptake and distribution of these materials as measured by NIRF; (2) to assess whether SWCNTs present in the diet can influence nutritional status through the measurement of expression of genes involved in nutrient processing. Data from this study will help to further our understanding of how dietary exposure to SWCNTs may influence uptake, distribution, and toxicity of these materials; essential knowledge for determining their behavior in aquatic systems.

## 2. Results and Discussion

One of the major challenges in the assessment of the environmental health and safety of SWCNTs is the lack of quick, low cost, and sensitive techniques for tracking the distribution and bioaccumulation of these materials *in vivo*. While techniques like TEM have been utilized to examine SWCNT uptake in aquatic organisms, they are extremely time consuming and lack the specificity to identify SWCNTs in tissue [6]. NIRF has emerged as a sensitive quantitative technique for tracking SWCNTs in water, sediment, and biological tissues [5,11,12,13]. Though the effectiveness of this technique in tracking these materials during oral exposure by gavage has been previously demonstrated [5], exposure through diet represents a more relevant scenario and affords the opportunity to examine novel mechanisms of sublethal toxicity.

**Figure 1:**
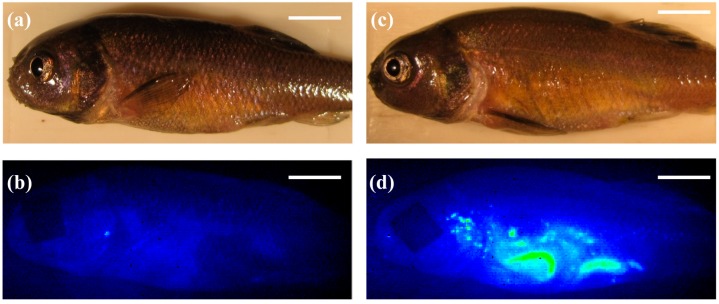
Near-infrared fluorescence (NIRF) imaging of fathead minnows (FHMs) fed single-walled carbon nanotubes (SWCNT) containing feed. Images of FHM fed a diet containing SWCNTs were collected throughout the study. The solid white line is a scale bar representing 5 mm. (**a**) Representative light image of control FHM; (**b**) Corresponding NIRF image of control FHM; (**c**) Representative light image of FHM exposed to SWCNT diet; (**d**) Corresponding NIRF image of FHM fed a SWCNT diet for 12 h. SWCNTs can be observed in live fish as indicated by increased fluorescence intensity of the pseudocolor.

**Figure 2:**
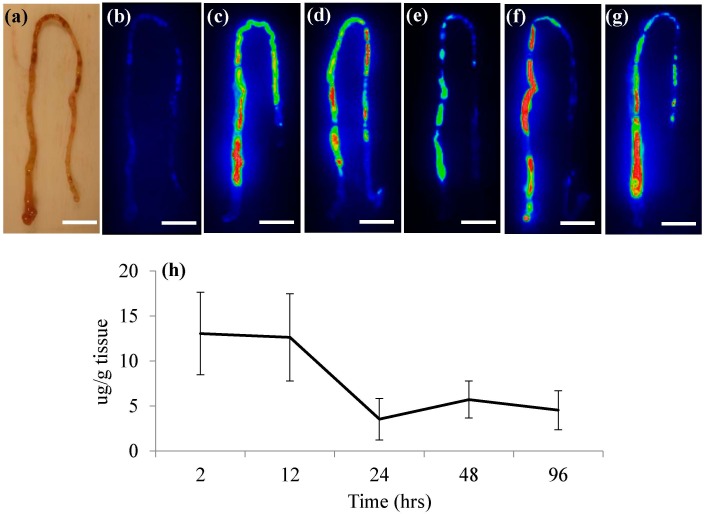
NIRF imaging and quantification of SWCNTs in FHM intestines. NIRF was used to track SWCNT movement in intestines throughout SWCNT exposures. The solid white line is a scale bar representing 5 mm. (**a**) Light image of typical FHM intestine with proximal portion oriented on the bottom left moving to distal portion; (**b**) NIRF image of control intestine; (**c**–**g**) NIRF images of representative intestines from FHM exposed to a SWCNT diet for 2, 12, 24, 48, and 96 h of feeding, respectively; (**h**) Quantitation of SWCNTs in FHM intestines using NIRF spectroscopy.

In the current study, fathead minnows (FHMs), *Pimephales promelas*, were exposed to SWCNT-containing diets for 96 h and NIRF was used to examine uptake of these materials. Exposure to these diets did not cause mortality or any observable morbidity in FHMs throughout the 96 h exposure period. This is similar to previous studies examining oral exposure of fish to SWCNTs that also found no overt toxicity [4,5]. Mixture of SWCNTs in the fish diet did not quench their fluorescence and within 2 h of the initial SWCNT feeding, fluorescence from the SWCNTs was observed in whole live fish using NIRF imaging (Figure 1d). The control diet also did not produce appreciable background fluorescence that would decrease the sensitivity of the NIRF imaging system (Figure 1b). Though SWCNT fluorescence could be observed in live fish throughout the exposure, individual organs were excised at time points for qualitative imaging and quantitative measurements of SWCNTs in FHM tissues. Both intestines and livers were removed from fish and imaged separately. NIRF imaging of FHM intestines indicated distribution of SWCNTs throughout the intestine for the entire duration of the exposure (Figure 2). Further quantification of SWCNTs in intestinal tissue using NIRF spectroscopy confirmed that feeding of a SWCNT diet remained fairly consistent throughout the exposure period (Figure 2h).

**Figure 3:**
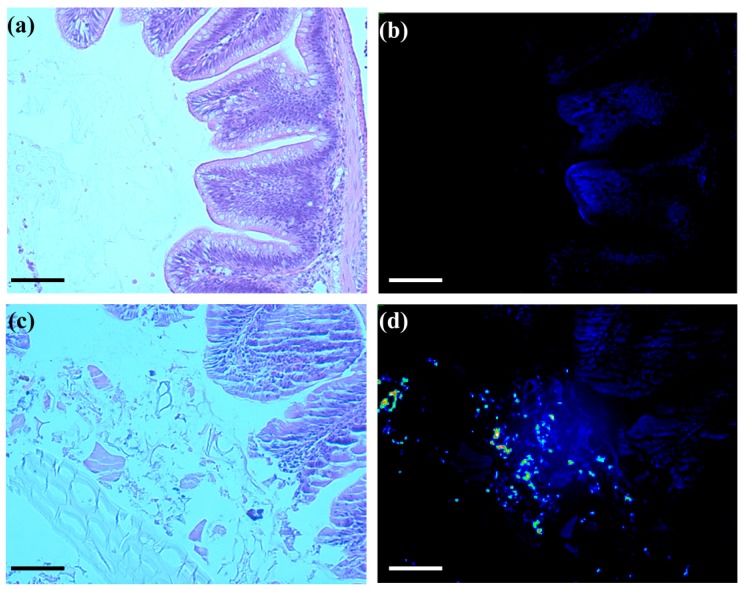
NIRF imaging of histological sections of FHM intestines. Histological sections of FHM intestines were examined to determine the localization of SWCNTs using NIRF. The solid lines is a scale bar representing 150 µm. (**a**) Representative light image of control FHM H&E stained intestinal cross section; (**b**) Corresponding NIRF image of control FHM intestinal cross section; (**c**) Representative light image of an intestinal cross section of FHM fed SWCNT diet; (**d**) Corresponding NIRF image of SWCNT fed intestinal cross section. SWCNT NIRF can be seen as increased fluorescence intensity of the pseudocolor. SWCNTs were only observed in the intestinal lumen with no evidence of association with or crossing the epithelia.

To examine the microscopic behavior of SWCNTs in the intestinal track of FHMs, NIRF microscopy was employed; a technique that has never been used in fish to date. Histological cross sections of exposed and unexposed FHM intestines were observed for SWCNT fluorescence using a microscope fitted with NIRF capability. While SWCNTs cannot be seen using light microscopy, NIRF microscopy allows for detection of the presence of SWCNTs in histological cross sections. Control cross sections exhibited low background fluorescence with no observation of significant fluorescence in the intestinal lumen or surrounding epithelia tissue (Figure 3b). Cross sections of SWCNT-exposed intestines revealed bright fluorescent SWCNTs among the intestinal lumen contents but no apparent association with intestinal epithelia or underlying tissue was observed (Figure 3d).

Contaminants that pass through the apical membrane of a fishes intestine and subsequently move through the basolateral membrane and into circulation are quickly transported to the liver which is involved in contaminant metabolism [25]. Therefore, it is likely that if SWCNTs were to pass through the intestinal epithelia they would find their way to the liver. Qualitative assessment of NIRF images of livers from FHMs exposed to SWCNT containing diets did not exhibit increased fluorescence when compared to control liver (data not shown). Additionally, quantitative analysis of livers using NIRF spectroscopy failed to detect SWCNTs in any of the fish livers (data not shown).

Using NIRF imaging of whole fish and individual organs, NIRF microscopy of intestinal cross sections, and NIRF spectroscopy of intestines and livers revealed that though FHMs readily consumed SWCNT containing feed, these materials stayed compartmentalized in the intestinal lumen and would likely be excreted. Studies have found a similar result when exposing FHMs to SWCNTs through oral gavage indicating that SWCNTs are not effectively absorbed in fish intestines [5]. In the aquatic invertebrate *Daphnia magna*, studies have shown that waterborne exposures to multi-walled carbon nanotubes (MWCNTs) and SWCNTs result in impaction of the intestine with materials but no appreciable uptake [6,26]. The presence of food may facilitate the absorption of organic contaminants in fish [27], but the results of this study indicate that this is probably not the case for SWCNTs. *In vitro* experiments have demonstrated that SWCNTs likely do not penetrate membranes directly but can be absorbed through endocytosis pathways [28] but the complexity of epithelia tissue and the presence of secretory mucous may preclude direct interaction with the intestinal brush border. Therefore, it is likely that any impact of oral exposure to SWCNTs on the gastrointestinal system will be through interaction with other constituents in the intestinal environment.

SWCNTs and MWCNTs have been shown to exhibit strong sorption capacity for a number of different proteins, lipids, and contaminants [17,18,19,29], which can decrease their bioavailability to aquatic organisms [30,31]. Based on our NIRF analysis it is unlikely that SWCNTs will be absorbed through the intestine however, interactions with nutritional components may render them unavailable for uptake, depleting essential nutrients needed by the fish for growth and reproduction. To test for potential effects of SWCNTs in the diet on nutrient uptake and processing, a suite of genes were selected that are known to be responsive to alterations in dietary nutrients. Before using these genes to test the effects of SWCNTs, they were first tested for their responsiveness to alterations in nutrient availability (starvation) as this is the first report to identify and quantify expression levels in FHMs. As the expression of these genes may not be equivalent throughout the intestine, we also probed their levels in defined sections: proximal, middle and distal.

**Figure 4:**
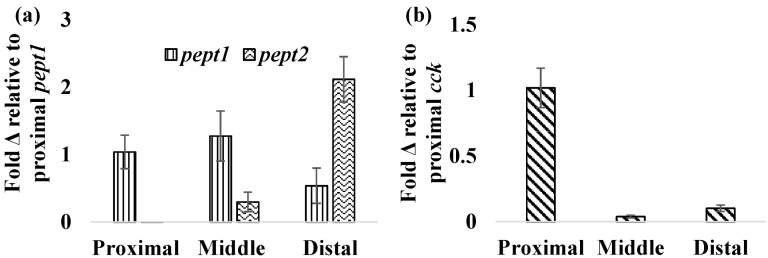
Relative expression of *pept1*, *pept2*, and *cck* in fish intestines. Expression of mRNA for *pept1*, *pept2*, *and cck* was measured in the proximal, middle, and distal intestines of normally fed FHMs to determine relative expression of these genes. (**a**) Relative mRNA expression of *pept1* and *pept2* throughout the FHM intestine. Expression is represented as the average ± standard error fold change from proximal *pept1* expression; (**b**) Relative mRNA expression of *cck* throughout the FHM intestine. Expression is represented as the mean ± standard error fold change from proximal *cck* expression.

The H^+^ dependent peptide transporters, *pept1* and *pept2*, are found along the apical membrane of the intestinal epithelium and are responsible for transporting digested dipeptides into intestinal tissues [32]. In normally fed FHMs, *pept1* was more highly expressed in the proximal and middle intestine with lower levels of expression found in the distal intestine (Figure 4a). Similar patterns were found in the European seabass with higher levels of *pept1* expression in the proximal intestine when compared to other regions and tissues [33]. Expression of *pept2* followed an opposite trend with the lowest expression observed in the proximal intestine, which increased distally with the highest expression in the distal intestine (Figure 4b).

**Figure 5:**
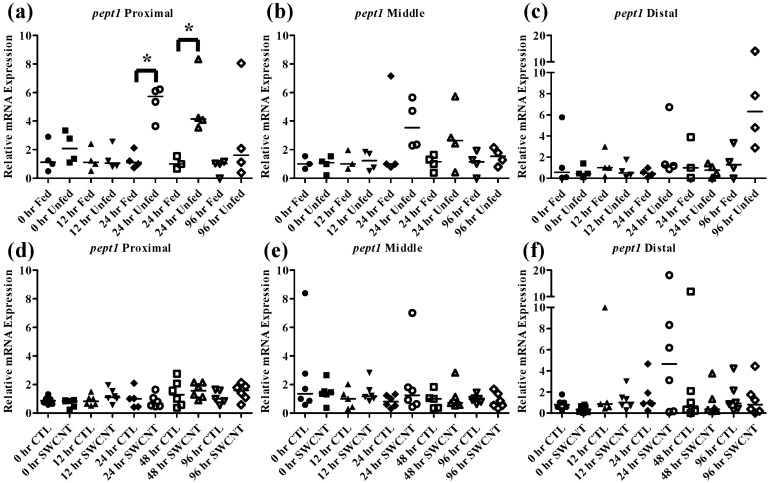
Intestinal *pept1* mRNA expression of fed, *vs.* unfed and control *vs.* SWCNT fed FHMs. Expression of *pept1* mRNA expression was measured in three intestinal sections of FHMs under different experimental conditions. Expression of individual replicate fish is presented relative to the corresponding control at that time point. Solid lines for each time point and treatment represents the median value. Statistical comparisons were made between treatments for each time point within each intestinal section and statistically significant values marked by an asterisk (*p* < 0.05). (**a**–**c**) Relative *pept1* mRNA expression in fed and unfed FHM proximal, middle, and distal intestines, respectively; (**d**–**f**) Relative *pept1* mRNA expression in proximal, middle, and distal intestines, respectively, of FHMs fed either control or SWCNT diets.

**Figure 6:**
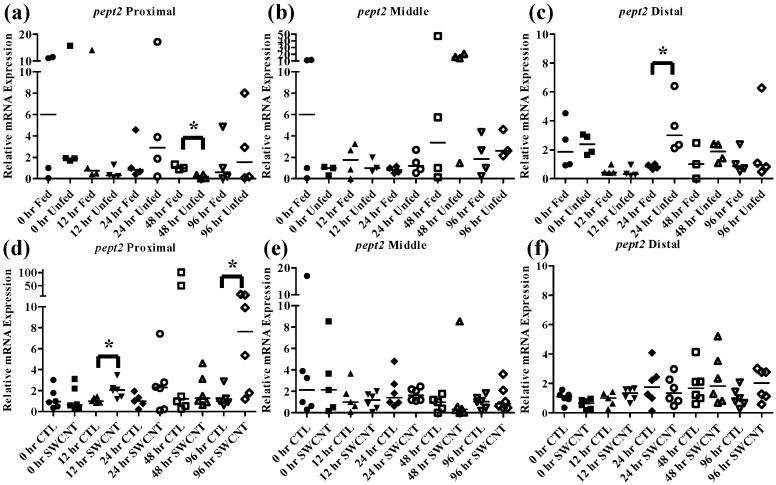
Intestinal *pept2* mRNA expression of fed, *vs.* unfed and control *vs.* SWCNT fed FHMs. Expression of *pept2* mRNA expression was measured in three intestinal sections of FHMs under different experimental conditions. Expression of individual replicate fish is presented relative to the corresponding control at that time point. Solid lines for each time point and treatment represents the median value. Statistical comparisons were made between treatments for each time point within each intestinal section and statistically significant values marked by an asterisk (*p* < 0.05). (**a**–**c**) Relative *pept2* mRNA expression in fed and unfed FHM proximal, middle, and distal intestines, respectively; (**d**–**f**) Relative *pept2* mRNA expression in proximal, middle, and distal intestines, respectively, of FHMs fed either control or SWCNT diets.

The effect of starvation on fish intestinal *pept1* expression has been examined in European sea bass and zebrafish, which exhibited opposing responses. In the European seabass, starvation for 7 days results in an increase in *pept1* expression but continued starvation for 35 days ultimately caused a decrease in expression [33,34]. This is likely due to fish increasing expression initially in an attempt to boost nutrient uptake followed by a decrease in expression to conserve energy during periods of extended starvation. In the zebrafish, starvation for 3 and 5 days caused decreased levels of *pept1* expression, which may be due to different feeding strategies and life histories of these fish [20,35]. It is also worth noting that like FHMs, zebrafish exhibit evolutionary loss of a stomach, which may also play a role in species differences. The function of the *pept2* transporter is not as well understood but one study found that during starvation of zebrafish, *pept2* expression initially decreases over a period of 6 h followed by increased expression up to 384 h [35]. In the current study, starvation of FHMs for 96 h caused increased expression of *pept1* in the proximal intestine (Figure 5a) by 24 h and high levels of expression were maintained until 48 h before returning to slightly above control levels by 96 h. While an increasing trend in *pept1* expression at 24 and 48 h (Figure 5b) was seen in the middle intestine, the values were not statistically significant due to high variability. Expression of *pept1* remained fairly constant in the distal intestine (Figure 5c) during regular feeding and starvation, likely due to overall lower expression in this region. Expression of *pept2* exhibited a unique pattern that was specific to the intestinal region. In the proximal intestine (Figure 6a), expression of *pept2* decreased by 48 h but returned to control levels by 96 h; but in the distal intestine (Figure 6c) expression of *pept2* increased at 24 h before returning to control levels by 48 h. While patterns of increasing and decreasing *pept2* expression in the intestine have been found before [35], a mechanistic explanation has yet to be elucidated. It was hypothesized that the differences found between fish species are due to dissimilarities in life histories and foraging strategies.

**Figure 7:**
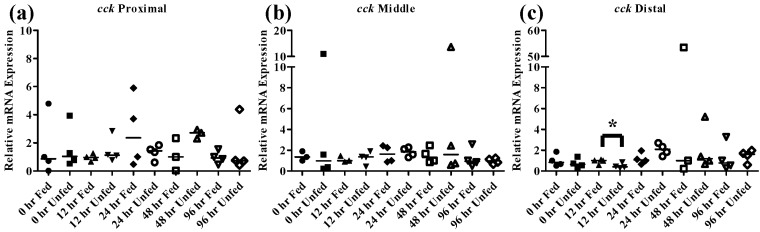

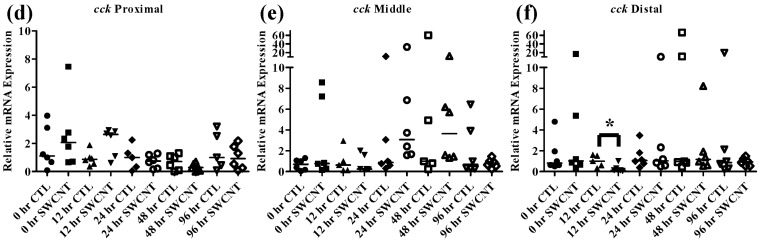
Intestinal *cck* mRNA expression of fed, *vs.* unfed and control *vs.* SWCNT fed FHMs. Expression of total *cck* mRNA expression was measured in three intestinal sections of FHMs under differing experimental conditions. Expression of individual replicate fish are presented as relative to the corresponding control at that time point. Solid lines for each time point and treatment represents the median value. Statistical comparisons were made between treatments for each time point within each intestinal section and statistically significant values marked by an asterisk (*p* < 0.05). (**a**–**c**) Relative *cck* mRNA expression in fed and unfed FHM proximal, middle, and distal intestines, respectively; (**d**–**f**) Relative *cck* mRNA expression in proximal, middle, and distal intestines, respectively, of FHMs fed either control or SWCNT diet.

The peptide hormone *cck* is responsible for controlling the release of digestive enzymes into the intestines of fish as well as playing role in nervous system control of appetite [36]. While *cck* has been detected in many tissues in fish most studies have focused on its distribution in the intestine. Numerous fish species including bowfin, bluegill, dorado, and rainbow trout, exhibit similar patterns of *cck* distribution throughout the intestine with high levels in the proximal intestine, varying levels in the middle intestine and low levels in the distal intestine [37,38,39]. Similar results were found in the FHMs used in this study where high expression of *cck* in the proximal intestine and lower levels found in the middle and distal intestine (Figure 4b) were observed. During periods of nutrient depletion, *cck* responses also appear to be conserved across species with starvation conditions causing decreased expression in winter flounder, zebrafish, and white sea bream [20,22,36]. While there was no significant *cck* response in unfed FHMs proximal and middle intestines (Figure 7a,b), the distal intestine (Figure 7c) did exhibit decreased *cck* expression after 12 h suggesting a signal to increase feeding due to decreased nutrient availability. Expression of *cck* returned to control levels by 24 h which may indicate a switch to conservation of energy used on foraging during periods of low food availability.

**Figure 8:**
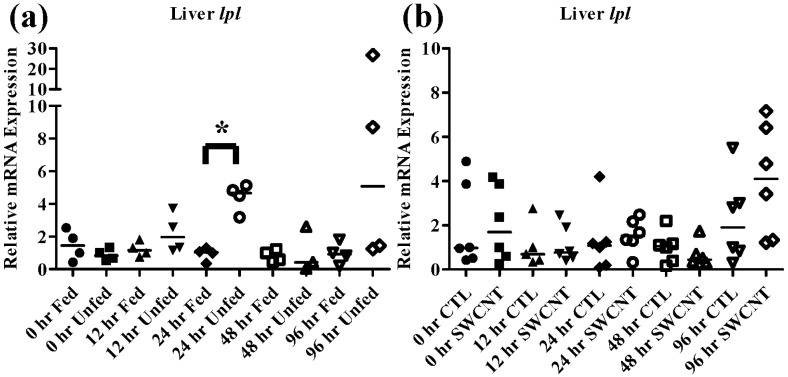
Liver *lpl* mRNA expression of fed, *vs.* unfed and control *vs.* SWCNT fed FHMs. Expression of *lpl* mRNA expression was measured in the livers of FHMs under different experimental conditions. Expression of individual replicate fish is presented relative to the corresponding control at that time point. Solid lines for each time point and treatment represents the median value. Statistical comparisons were made between treatments for each time point within each intestinal section and statistically significant values marked by an asterisk (*p* < 0.05). (**a**) Relative *lpl* mRNA expression in fed and unfed FHM livers; (**b**) Relative *cck* mRNA expression in livers of FHMs fed either control or SWCNT diets.

Lipoprotein lipase (*lpl*) is an enzyme responsible for digestion of circulating lipids and though it is typically found in adipose and other tissues in mammals, fish exhibit *lpl* expression and activity primarily in the liver. During periods of starvation expression of *lpl* has been shown to increase as decreases in circulating insulin gives way for use of lipids as a primary energy source, which has been demonstrated in numerous fish species including red sea bream and tilapia [23,40]. In the current study FHMs that were starved for 96 h exhibited increased *lpl* expression by 24 h, which returned to control levels by 48 h (Figure 8a).

To determine whether interactions of SWCNTs and intestinal nutrients can cause nutrient depletion conditions in FHMs, fish were exposed to a SWCNT containing diet for 96 h and *pept1*, *pept2*, and *cck* were measured in the proximal, middle, and distal intestines as well as *lpl* being measured in livers. While the SWCNT diet did not impact *pept1* expression in any of the intestinal regions (Figure 5d–f), *pept2* increased significantly at 12 h and 96 h in the proximal intestine (Figure 6d–f). While this was quite different, and in fact opposite, from what was seen during starvation conditions, it does indicate that SWCNTs may be impacting the availability of nutrients in FHMs. Starved zebrafish have also been shown to exhibit increased expression of *pept2* which may be an indication of an attempt to increase nutrient uptake during periods of decreased nutrient availability [35]. But the fact that *pept2* expression alternates between increased expression and basal expression levels highlight the transient nature of these peptide transporters in fish which have been shown to exhibit differential expression patterns depending on species and duration of starvation [20,33,34,35]. While a comprehensive explanation for this activity has not been elucidated it is possible that individual species have evolved different strategies to deal with times of decreased nutrient depletion.

As discussed above, *cck* has been shown to decrease during starvation periods, a response that has been shown to be conserved in numerous fish species [20,22,36]. During exposure to SWCNTs, an identical response was observed in FHMs starved for 12 h, as expression of *cck* in the distal intestine was significantly decreased (Figure 7d–f). This may also indicate that FHMs were experiencing decreased nutrient availability and uptake due to interactions of SWCNTs with dietary nutrients. But expression of *lpl* was not altered during SWCNT exposure, which could indicate preferential sorption of proteins in the diet or that a significant portion of lipids in the diet were still bioavailable.

**Figure 9:**
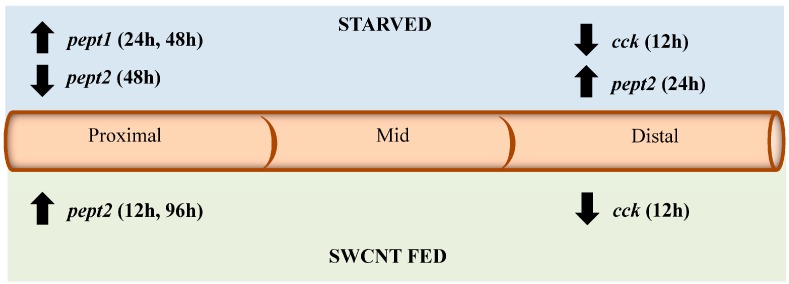
Summary of impacts of starvation and SWCNT diet on *pept1*, *pept2*, *cck*, and *lpl* expression. The diagram shows the direction of expression (arrows) and time (in parenthesis) for each gene in the proximal, middle and distal sections of the intestine for both starved and SWCNT fed conditions.

Impacts on *pept1*, *pept2*, *cck*, and *lpl* from the fed *vs.* unfed and SWCNT feeding studies are summarized in Figure 9. Overall, starvation of FHMs causes similar responses in *pept1*, *pept2*, *cck*, and *lpl* expression as seen in a number of other fish species. It was hypothesized that SWCNT interactions with nutritional components in the FHM diet would cause similar responses to a fish lacking essential nutrients. While the presence of SWCNTs in the diet had no impact on *lpl* and *pept1* expression they did cause impacts on expression of *pept2* and *cck*. Interestingly, these impacts were seen in sections of the intestines where these genes are normally expressed at relatively low levels which may indicate that impacts of the SWCNTs are subtle and perhaps not significant enough to impact sections of the intestine that are primarily responsible for nutrient processing. Yet these results do represent a novel pathway through which SWCNT may indirectly cause sublethal toxicity in aquatic organisms even if the intestinal tract does not absorb them. Impaction of the intestines of *Daphnia magna* by SWCNTs and MWCNTs has also been implicated as a mechanism through which these materials may alter an organism’s ability to efficiently absorb nutrients [6,26,41]. But further studies are needed to consider the long-term phenotypical responses that subtle indirect effects of SWCNTs on nutrient processing may be impacting.

## 3. Experimental Section

### 3.1. Single-Walled Carbon Nanotube Suspension Preparation and Characterization

Chirally enriched semi-conducting grade (SG65) SWCNTs were generously donated by SouthWest Nano-technologies (Norman, OK, USA) in dry form. For addition to diets for feeding studies, SWCNT suspensions were prepared as described by Bisesi *et al*. [5]. Briefly, 1 mg of dry SWCNTs were added to 1 mL of 0.5% gum arabic, vortexed, and ultrasonicated. Resulting suspensions were centrifuged to remove unsuspended aggregates and the supernatant was used for subsequent diet preparations. Final concentrations of suspensions were measured using NIRF using previously described methods [13]. Characterization of these suspensions has previously been reported in Bisesi *et al*., 2014 [5] which showed an average particle size of 132 nm (DLS), moderately compact aggregates with a fractal dimension of 2.2–2.3 (SLS), and less than 5% *w*/*w* metal catalyst with approximately 3.8% molybdenum and 0.93% cobalt leaching from these materials when in suspension. DLS and SLS data indicate that these suspensions are very stable and the measured particle characteristics are maintained for at least a week. These results are consistent with previous observations [42,43,44].

### 3.2. Single-Walled Carbon Nanotube Diet Preparation

Diets for the feeding experiments were prepared by grinding 4 mm Skretting Salmon 45–19 (45% protein, 19% fat, 3% fiber, Tooele, UT, USA) pelleted fish feed to a fine powder using a coffee grinder. Five grams of ground feed was mixed with 2 mL of SWCNT stock in a 10 mL volumetric flask and filled to slightly below 10 mL with 0.5% gum arabic. The solution was then sonicated at 10% amplitude using a Branson Sonifier 450 fitted with a 1/8" diameter microtip probe for 30 s to ensure homogenization of the SWCNTs in the feed. Unflavored gelatin (590 mg) was then added to the solution and the volume brought up to 10 mL. The mixture was then inverted several times to dissolve the gelatin and poured into a 10 × 8.5 cm hexagonal weigh boat, which was then placed in the refrigerator for solidification. After 1 h solidified feed was removed from the weigh boat, the entire batch of feed was weighed, and cut into strips. Control feed was prepared using the same methods with the SWCNT suspension being replaced with 0.5% gum arabic.

### 3.3. Primer Design and Validation

Primers for the fathead minnow (*Pimephales promelas*) housekeeping gene *rpl8* were chosen from Overtruf *et al*. [45] as they were previously validated. Forward and reverse primers for *cck* were chosen from Popesku *et al*. [46] and independently validated in house using the methods described below. Primers for *pept1*, *pept2*, and *lpl* have not been previously published for fathead minnows, therefore new primers were designed for this study. Annotated contigs for these genes were obtained from the TGI database of the fathead minnow transcriptome [47] and used to design primers for quantitative PCR. Sequences were entered in Integrated DNA Technologies’ PrimerQuest (IDT, Coralville, IA, USA) and selected based on acceptable melting temperatures, GC content, amplicon size, homodimers, and heterodimers. Lyophilized primers received from IDT and were dissolved in RNase/DNase free water to a stock concentration of 100 µM and further diluted to 10 µM for PCR reactions. To determine the specificity and efficiency of primer sets, PCR amplicons were cloned to create template serial dilutions for validation. RNA pools from untreated fathead minnow intestine and liver tissues were extracted, DNase treated, and reverse transcribed as detailed in section 3.8. PCR reactions were prepared as follows: 12.5 µL of PCR master mix (Promega, Madison, WI, USA), 1 µL (10 µM) forward primer, 1 µL (10 µM) of reverse primer, 1 µL of template, 9.5 µL RNase/DNase free water. PCR was performed in an Applied Biosystems Veriti Thermal Cycle (Life Technologies, Grand Island, NY, USA) using the following cycling parameters: 1 cycle, 95 °C for 5 min; 40 cycles, 95 °C for 30 s, 58 °C for 30 s, 72 °C for 1 min; 1 cycle, 72 °C for 10 min; hold at 4 °C. The resulting PCR product was loaded with 2 µL of loading dye and run on a 2% agarose gel stained with ethidium bromide (200 µg/L, Fisher Bioreagents, Pittsburg, PA, USA). Gels were run at 100 V for 45 min to achieve band separation and compared to concurrently run DNA ladder for size verification (Fisher Bioreagents exactgene mid range plus). Primer sets that produced single products of the correct size were excised from gels and extracted for cloning using the QIAquick Gel Extraction Kit (Qiagen, Germantown, MD, USA) following the supplied protocol. Resulting amplicons were ligated into the pGEM-T Easy vector (Promega) followed by transformation into chemically competent JM109 cells (Promega) using manufacturer provided protocols. Bacterial clones containing ligated pGEM-T Easy vectors with amplicon inserts were selected for by resistance to ampicillin containing LB agar and confirmed by sequencing. Clones containing the correct inserts were then grown in LB media and vectors were extracted and purified using the Endofree Plasmid Maxi Kit (Qiagen) following the manufacturers protocol. Purified templates for each primer set were then 10 fold serially diluted in RNase/DNase free water for use in primer qPCR validation.

Template standard curves for *pept1*, *pept2*, *lpl*, and *cck* ranged from 5 to 5 × 10^−7^ ng. Reactions for quantitative PCR were prepared as follows: 10 µL of SYBR^®^ Select Master Mix (Life Technologies), 1 µL forward primer (18 µM), 1 µL reverse primer (18 µM), 1 µL template, 7 µL RNase/Dnase free water. Quantitative PCR reactions were performed using a MyiQ™ Real-Time PCR Detection System (Bio-Rad, Hercules, CA, USA) using the following parameters: 1 cycle, 95 °C for 3 min; 40 cycles, 95 °C for 10 s, 58 °C for 1 min. Using the iQ™5 optical system software (Bio-Rad), threshold cycles were plotted against template concentrations to determine the efficiency and correlation of each primer set which are given in Table 1. Finally, melt curve analysis was used to ensure primers produced single products. Melt curve cycles were from 55 °C to 95 °C increase 0.5 °C every 30 s and plotted in the iQ™5 software. Only primer sets with efficiencies between 95% and 105% and single melt curve products were used for qPCR analysis. Final primer sets and efficiencies are provided in Table 1.

**Table 1:** Primers used for measuring mRNA expression in fathead minnows.

Gene	Forward (5'-3')	Reverse (5'-3')	Efficiency	Source
*pept1*	ACACCGCAGTAAGCAATACC	ACCTTCAGTGCCATCTTTACC	103.3%	In house
*pept2*	CAGTGATTGGTCTGGTCCTTAT	TCGCCTTTCGTCTGTATTCTC	102.5%	In house
*cck*	ACCAGCCTCACCCTCAAA	AAATCCATCCAGCCCACA	96.5%	[46]
*lpl*	CTGTGACCTCCAGAACACTATG	GAGTCGATGAACAGGTGGATG	103.9%	In house
*rpl8*	CATACCACAAGTACAAGGCCAAGA	ACCGAAGGGATGCTCAACAG	97.0%	[45]

### 3.4. Nutrient Depletion Study for Testing Gene Responses

All fish studies were conducted with approval from the University of Florida Institutional Animal Care and Use Committee. Fathead minnows were obtained from in house cultures at the University of Florida Aquatic Toxicology Core Laboratory. The two treatments tested were fish starved for 96 h and fish fed the diet described above containing gelatin vehicle for 96 h. Twenty fathead minnows (mass: 2.72 ± 0.84 g; length: 59.8 ± 6.6 mm) for each treatment were placed in 37 L aquariums which were maintained as flow through systems throughout the experiments. Both fed and starved fish were acclimated to the feed as described in Section 3.2 for 48 h before initiating fed and starved treatments. At time 0, fathead minnows were either fed or starved for the first time. Feedings took place by weighing food equivalent to 3% of the total biomass/day, which was split between two daily feedings. Gelatin food was diced into fine pieces with a razor blade to ensure the fish could consume them. Fish consumed all feed within 5 min of administration. Feed mass was adjusted daily to account for the number of individual fish left in the tanks following time points. At 0, 12, 24, 48, and 96 h, 4 fish from each treatment were euthanized by immersion in 100 mg/L Tricaine-S (Western Chemical Company, Ferndale, WA, USA) until opercular movement ceased followed by cervical dislocation. Euthanasia was conducted 2 h after the terminal feeding event to ensure that digestion was occurring. Fish were weighed and measured and intestines and livers were excised and snap frozen in liquid nitrogen followed by storage at −80 °C for future analysis.

### 3.5. Single-Walled Carbon Nanotube Feeding Experiments

Fathead minnows were obtained from in house cultures at the University of Florida Aquatic Toxicology Core Laboratory. The two treatments tested were control gelatin feed and SWCNT gelatin feed (50 µg SWCNTs/g food) which were administered at a rate of 3% of the total tank biomass/day split between two different feedings. Gelatin food was diced into fine pieces with a razor blade. Feed mass was adjusted daily to account for the number of individual fish left in the tanks following time points and all feed was consumed within 5 min of administration. Individual 37 L aquaria were used for each time point and treatment with 10 minnows in each aquarium. At each time point (0, 12, 24, 48, and 96 h) minnows for each treatment were euthanized by immersion in 100 mg/L Tricaine-S until opercular movement ceased followed by cervical dislocation. Euthanasia was conducted 2 h after the terminal feeding event. Fish were weighed, measured, and livers and intestines were excised. Four of the fish for each treatment and time point were used for NIRF imaging and quantification which is described in Section 3.7. From the remaining 6 fish livers were excised and snap frozen in liquid nitrogen followed by storage at −80 °C for qPCR analysis. Intestines were also removed and split into proximal, middle, and distal sections. From each section half the sample was saved for qPCR by snap freezing in liquid nitrogen and storage at −80 °C while the other half was preserved in 10% phosphate buffered formalin (Fisher Scientific, Pittsburg, PA, USA) for histology (Section 3.6) and NIRF imaging (Section 3.7).

### 3.6. Histology

Sections of fathead minnow intestines were preserved for histological examination of the presence of SWCNTs in the intestinal lumen as well as any interactions and absorption through the intestinal epithelium. Following euthanasia, whole intestines were removed from fish and separated into proximal, middle, and distal sections. Individual sections were preserved in 10% phosphate buffered formalin for at least 24 h before processing. Preserved intestinal sections were embedded in paraffin wax, sectioned at 5 um, and stained with hematoxylin and eosin for general tissue-level observations and NIRF imaging.

### 3.7. Near-Infrared Fluorescence Imaging and Quantitation

NIRF imaging was used to track SWCNT distribution in fathead minnows during feeding studies. The custom system used for this analysis is described in detail in Bisesi *et al*. [5]. The system uses a one second pulse from 808 nm laser set to 5 W to excite fish tissues. Fluorescent emission from SWCNTs in the near-infrared is selected for by dichroic mirrors and filters and captured by a two dimensional infrared InGaAs array detector cooled to −100 °C (Princeton Instruments, Trenton, NJ, USA). The resulting image is captured by Princeton Instruments WinSpec Software. This technique has been shown to exhibit high selectivity for SWCNTs and low background fluorescence in biological tissues. At each time point 4 fish from each treatment were imaged using this system including images of the whole fish, the whole fish with the left filet removed, and excised intestines and livers. Images were qualitatively assessed between treatments to identify any evidence of uptake through intestines and any presence of SWCNTs in the liver. Sampled were then snap frozen in liquid nitrogen and stored at −80 °C for quantitative measurement of SWCNTs described below.

In addition to gross examination of intestinal tissue using NIRF, histological sections were also examined for microscopic evidence of SWCNT uptake through intestinal epithelia. The NIRF imaging system was coupled to a Nikon Eclipse Ti-U inverted fluorescence microscope by connecting the Princeton Instruments 2 dimensional array detector to one of the high sensitivity imaging ports on the scope using a 1× c-mount adaptor. Samples were excited by a fiber coupled solid state laser (735 nm, 450 mW, B&WTek, Newark, DE, USA) which was introduced to the microscope through a manual total internal reflection fluorescence (TIRF) adaptor and aligned to the center of the slide to achieve epifluorescene of the sample. Fluorescence in the near-infrared was selected for by a filter cube mounted dicroic mirror (<875 nm transmission, Semrock Inc, Chicago, IL, USA) and a long pass filter (900 nm, Chroma Technology Corp, Bellows Falls, VT, USA). Glass mounted intestinal sections were imaged using NIRF to examine for evidence of SWCNTs associated with the epithelia or their retention in the lumen.

Whole intestines and livers that were imaged with NIRF were quantified using NIRF spectroscopy following a previously published method [5,13]. Tissues were suspended in 1 mL of 2% sodium deoxycholate (SDC, Fisher Scientific) and ultrasonicated (Branson Sonifier 450) using a 1/8" microtip at 50% amplitude for 10 min on ice. The resulting suspension was transferred to a quartz cuvette and measured on an Applied NanoFluorescence NS1 spectrofluorometer (Applied NanoFluorescence, Houston, TX, USA). A fix point matrix matched standard addition of SG65 SWCNTs to unexposed fathead minnow intestines was used to account for matrix effects and recovery.

### 3.8. Quantitative PCR

Total RNA was extracted from intestine and liver samples from the nutrient depletion and SWCNT feeding studies using a modified method from Mehinto *et al*., 2014 [48]. In brief, tissues were homogenized in RNA Stat-60 (Tel-Test, Friendswood, TX, USA) using a handheld rotary homogenizer followed by addition of chloroform. Aqueous RNA was precipitated with ethanol, re-dissolved in RNAsecure (Life Technologies, Grand Island, NY, USA), and measured using a Thermo Scientific NanoDrop 1000 spectrophotometer (Thermo Fisher Scientific, Wilmington, DE, USA). Samples were then DNase treated (Turbo DNA-free kit, Life Technologies, Grand Island, NY USA) and reverse transcribed to cDNA (Reverse Transcription System, Promega, Madison, WI, USA). Expression of *pept1*, *pept2*, *cck*, and *rpl8* was measured in intestinal preparations and expression of *lpl* and *rpl8* was measured in liver preparations for each exposure experiment. Quantitative PCR reactions were prepared and run using the cycling parameters described above in Section 3.3. Relative fold change in mRNA expression from controls was analyzed using the 2^−ΔΔCt^ method.

### 3.9. Statistical Analysis

Statistical analysis was conducted in GraphPad Prism 5 (GraphPad Software Inc, La Jolla, CA, USA). All mRNA expression data was tested for normality using the Kolmogorov-Smirnov test and homogeneity of variances using the F tests of equality of variances. Expression of controls and treatments within each time point were compared by unpaired *t*-tests. Time points that were found to non-normal distributions and/or unequal variances were compared using nonparametric *t*-tests (Mann-Whitney test). *p* values < 0.05 were considered significant.

## 4. Conclusions

NIRF has been shown to be a powerful tool in the assessment of uptake and distribution of SWCNTs in aquatic organisms. In the current study, NIRF imaging, microscopy, and spectroscopy showed that the presence of food does not facilitate the absorption of SWCNTs through the intestines of FHMs. But the potential for interactions between SWCNTs and nutrients in the food led to the examination of sublethal impacts on expression of nutrient processing genes. Starved FHMs exhibited similar responses in expression of *pept1*, *pept2*, *cck*, and *lpl* to numerous other fish species. But SWCNTs only caused impacts on *pept2* and *cck* in regions of the intestine where they exhibit low expression indicating subtle effects on nutrient transport. These sublethal impacts represent a novel pathway by which SWCNTs may indirectly impact aquatic organisms, even if these materials are not readily absorbed through the intestine. Further studies are needed to determine whether these subcellular responses will result in adverse phenotypes with population level consequences.
